# Preparation and Application of Nanocomposite Thin-Film Temperature Sensor during the Milling Process

**DOI:** 10.3390/ma15207106

**Published:** 2022-10-13

**Authors:** Yunxian Cui, Haoyu Wang, Kaidi Cao, Qunli Zhou, Wanyu Ding, Junwei Yin

**Affiliations:** 1 School of Mechanical Engineering, Dalian Jiaotong University, Dalian 116028, China; 2 West-East Gas Transmission Branch of State Petroleum and Natural Gas Pipeline Network Group Co., Ltd., Nantong 226000, China; 3 School of Materials Science and Engineering, Dalian Jiaotong University, Dalian 116028, China

**Keywords:** thin-film thermocouples, integrated wire substrates, tool-chip contact area, continuous temperature measurement

## Abstract

During the titanium alloy milling process, high temperatures in the tool-chip contact area will affect the tool life and precision of titanium alloy machining. Therefore, it is essential to measure the temperature of the tool-chip contact area continuously. In this paper, a finite element simulation model of the milling process was established using ABAQUS2020 to obtain the highest temperature location in the tool-chip contact area when milling titanium alloy. The integration of the wire with the alumina ceramic substrate formed an integrated wire substrate. Furthermore, NiCr, NiSi, and SiO_2_ films were deposited on the substrate sequentially using the DC pulsed magnetron sputtering technique. Finally, its microscopic morphology and static and dynamic performance were tested. The results show that the developed thin-film thermocouple temperature sensor has a Seebeck coefficient of 40.72 μV/°C and a dynamic response time of 0.703 ms. The application of the sensor to our titanium alloy milling experiments showed that the sensor can monitor the transient temperature in the tool-chip contact area, and its temperature measurement performance showed no detrimental effect from wearing. The effect of each milling parameter on the milling temperature was analyzed using ANOVA, and a regression model with an R-sq of 96.76% was obtained for the milling temperature.

## 1. Introduction

With the rapid development of the modern aviation industry, higher requirements are put forward for the endurance and load-bearing capacity of aviation equipment [1]. Using advanced materials is one of the fundamental ways to achieve weight reduction and efficiency. Titanium alloy has the advantages of low specific gravity, high specific strength, and high compatibility with carbon-brazed dimensional composites and has been widely used in high-end equipment in the aviation field [2]. However, titanium alloy is a material that is typically difficult to machine, and the tool-chip contact region is the highest temperature point during machining. The strong adhesion between the tool-chip and tool-workpiece interfaces at this location [3] makes it more challenging to measure the maximum temperature of titanium alloys. The thermal conductivity of titanium alloy is low compared to other metals; therefore, if the cutting heat of titanium alloy is not monitored and controlled, it will lead to faster tool wear, poor workpiece machining quality, and low surface accuracy, which will seriously affect the tool’s life span [4,5,6]. Therefore, to achieve the efficient and precise machining of aerospace titanium alloy parts, it is essential to accurately and, in real-time, continuously measure the temperature in the tool-chip contact area during machining.

Milling, one of the most basic and common ways to advance machine materials, has been the focus of research in titanium alloy machining. This high rotational speed of the tool and the destructive machining environment make the titanium alloy milling temperature more difficult. In recent years, numerous scholars have conducted much research on the measurement of milling temperatures [7,8], and specific results have been achieved. Emilios Leonidas et al. [9] summarized the main measurement methods for cutting temperature, especially in high-speed machining applications (milling and drilling). They classified the measurement methods mainly into infrared and thermocouple methods. This is also the most dominant test method used by scholars for measuring milling temperature.

The infrared radiation method uses a thermal imaging infrared camera to determine the temperature distribution of the tool or workpiece during the machining process. Baralić J Č et al. [8] measured the end milling temperature, which was used for subsequent modeling and optimization, and they used an infrared camera for the measurements, placing the infrared camera 500 mm away from the milling machine. They experimentally concluded that the temperature at the cutting edge of the milling blade was not the highest during the milling process. The temperature at the front tool face, where the tool-chip contact was located, was the highest.

The infrared radiation method is a non-destructive way to measure the milling temperature and has a high response time. However, it can only estimate the surface temperature and is subject to more significant interference from environmental factors, especially in the high-speed rotating milling process. The infrared camera lens is easily obscured by the chips on the workpiece, thus affecting measurement accuracy.

The thermocouple method includes the natural thermocouple method, the semi-artificial thermocouple method, and the artificial thermocouple method. The natural thermocouple method is the most common method used by early researchers to measure milling temperature. The tool and the workpiece are two different materials that form a thermocouple to measure the temperature of the machining process. Kitagawa et al. [10] measured the temperature of turned and milled titanium alloys (without coolant) via the natural thermocouple technique and explored the tool life of titanium alloy end faces that were machined by both methods. The semi-artificial thermocouple method is a metal wire embedded in the tool or workpiece. Two different conductors form a closed circuit from the tool or workpiece and the metal wire to form a thermocouple. Baohai Wu et al. [11] proposed a method for predicting the temperature of mill ends based on an analytical model. The method’s accuracy was verified by finite element models and temperature measurement tests. Their tests were conducted by dividing the workpiece into two parts separated by an insulating layer along the milling direction, fixing three NiCr wires in the workpiece through an adhesive, and measuring the temperature of the milling, and both the experimental measurements and the data from the finite element model follow the same trend. The artificial thermocouple method involves embedding a thermocouple in the workpiece or tool to measure the milling temperature. Alexandre Il et al. [12] used cutting temperature as an indicator in order to optimize the cutting dosage for milling end thin-walled aluminum alloy parts, and a filament thermocouple with a response rate of 20 ms was embedded within the pre-drilled hole in the workpiece at different positions along the milling direction. The results showed that the optimal milling conditions were the low depth of cut, high cutting speed, and a minimum of 0.005 inches per feed. Since milling is a process that destroys the workpiece, it is also necessary to avoid damage to the sensor when embedding the thermocouple in the tool.

Thorsten Augspurger et al. [13], in order to achieve continuous temperature measurement, embedded a 0.5 mm diameter K-type thermocouple wire in a blind hole 2 mm from the cutting edge, thus avoiding the problem of easily damaging the temperature-sensing end of the wire thermocouple after suffering frictional wear, and used a wireless FM/PCM transmitter to transmit temperature data. The temperature measurement device was tested by milling titanium alloy with a sampling rate of 6620 Hz and a sensor response time of 0.2 s.

Similarly, Muhammad Rizal et al. [14] inserted a K-type thermocouple wire between the milling insert and the tool holder, thus preventing the sensor from wearing out and obtaining the temperature during the milling of ductile iron. Although the thermocouple is less expensive, the response speed is slow, especially in the high-speed tool rotation, which cannot capture rapid temperature changes [9].

A thin-film thermocouple is a new type of transient temperature sensor based on a thermoelectric conversion mechanism. It has the advantages of being small in size and having a convenient design and can be integrated directly into the tool to measure the transient temperature in the tool-chip contact area during machining. Jiang Li et al. [15] used a laser to etch 10 micro-grooves on the front face of a material WC-Co turning tool and then deposited an Al_2_O_3_ insulating film, Cr film (in turn), and a WC-Co material with a Cr film to form a sensor array; the response time of the sensor was experimentally measured to be about 10 ms. Tianxiang Li et al. [16] used laser etching to etch two 100 μm-deep microgrooves on the rear tool surface of a turning tool and deposited NiCr, NiSi functional films, and Si3N4 protective films sequentially into the microgrooves for cutting experiments with hardened AISIO2 tool steel bars; the results of the study showed that the sensor’s sensitivity was 14.4 μV/°C. Due to the complexity of milling operations, there are only a few types of research on thin-film thermocouple deposition in milling tools. Sinan Kesrikliogiu et al. [7] used a magnetron sputtering process to deposit a K-type thermocouple film directly onto the front tool face of a milling cutter and transmitted the temperature signal to an acquisition system through a slip ring to measure the milling temperature in the tool-chip contact zone of an AISI 4130 alloy steel plate. However, because the thermosensitive end of the thin-film thermocouple comes into direct contact with the tool-chip contact area, the inevitable contact friction during milling will damage the membrane thermocouple, thus damaging the thin-film thermocouple and preventing it from continuously obtaining the temperature of the tool-chip contact area.

The traditional milling temperature test method has many shortcomings in sensitivity, response time, and sensor arrangement. As a new test method for cutting temperature, the thin-film thermocouple still has some problems, such as an unsafe lead film connection and abrasion resistance. This paper uses ABAQUS for the thermal simulation of the titanium alloy milling process and obtains the highest temperature point in the tool-chip contact area when milling titanium alloy. We proposed a new thin-film thermocouple structure that can be worn and closely matched with the carbide milling cutter blade using static molding technology to integrate the compensation lead and the sensor substrate. On this basis, the thin-film thermocouple temperature sensor was prepared by magnetron sputtering technology, and the microscopic morphology of each layer of the film was characterized. Moreover, the new thin-film thermocouple temperature sensor’s accuracy, stability, sensitivity, and repeatability were studied. Finally, the developed thin-film temperature sensor was applied to titanium alloy milling, and the transient temperature change curve of the tool-chip contact area and the wear condition of the thin-film temperature sensor were obtained.

## 2. Titanium Alloy Milling Simulation

### 2.1. Titanium Alloy Milling Modeling

**Figure 1:**
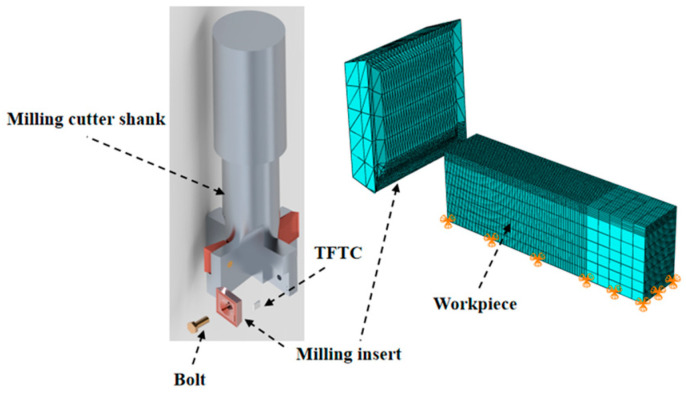
Simulation model and meshing of titanium alloy milling.

In this paper, titanium alloy is milled with a T-shaped milling cutter as the object of the study. The T-shaped milling cutter mainly contains a shank, milling cutter blade, and fixing bolt. Each milling cutter is equipped with four milling cutter blades, which are fixed on the milling cutter shank via a bolt. The milling cutter blade was made of carbide (model CPMT1604), the back angle of the milling cutter blade was 11°, and the geometry size was 16 mm × 16 mm × 4 mm. In order to improve the efficiency of the simulation calculation, the milling model was simplified to the cutting of the workpiece by the milling cutter, and the workpiece material was TC4 titanium alloy. The titanium alloy workpiece was 50 mm × 10 mm × 30 mm. The model was meshed using ABAQUS with a gradient mesh, and the mesh was denser in the area where the tool was in contact with the workpiece. The unit size was 0.3 mm and 1.5 mm, respectively. The number of units was 10,899 and 41,400, respectively, and the friction coefficient between the tool and workpiece was 0.15. The completed titanium milling model is shown in Figure 1. The contact mode was set to “face-to-face” contact between the tool and the workpiece. The tool was set as a rigid body, and the cutting speed was 188.40, 150.72, and 113.04 m/min, with a feed of 200 mm/min, and the cutting depth was 0.5 mm. The simulation calculation was carried out respectively. The initial temperature of the model was 20 °C. In this paper, the Johnson–Cook model, which can describe material strain variation, was chosen as the intrinsic model to define the material properties of titanium alloy. The expressions are as follows [17].

(1) $$\sigma =[A+B\varepsilon^{n}][1+C\ln(\frac{\varepsilon^{\prime }}{\varepsilon_{0}{}^{\prime }})][1+{\left(\frac{T-T_{0}}{T_{\mathrm{melt}}-T_{0}}\right)}^{m}]$$

where:

σ—Material flow stress;

*A*—Yield strength;

*B*—Modulus of hardening;

ε—Plastic deformation;

*n*—Hardening factor;

*C*—Strain rate sensitivity factor;

$\varepsilon^{\prime }$—Strain rate;

$\varepsilon_{0}^{\prime }$—Reference strain rate;

T—Calculated temperature of cutting material;

$T_{\mathrm{melt}}$—Melting temperature of cutting material;

$T_{0}$—Room temperature;

m—Thermal softening coefficient.

Among them, the parameters related to the JC model of titanium alloy material are yield strength (*A*), hardening modulus (*B*), strain rate sensitivity coefficient (*C*), hardening coefficient (*n*), and thermal softening coefficient (m). In addition, in order to calculate the temperature of the milled titanium alloy, the values of the JC principal and the remaining related parameters are shown in Table 1. The temperature distribution in the tool during the titanium alloy milling process was successfully simulated.

### 2.2. Analysis of the Titanium Alloy Milling Simulation Results

**Figure 2:**
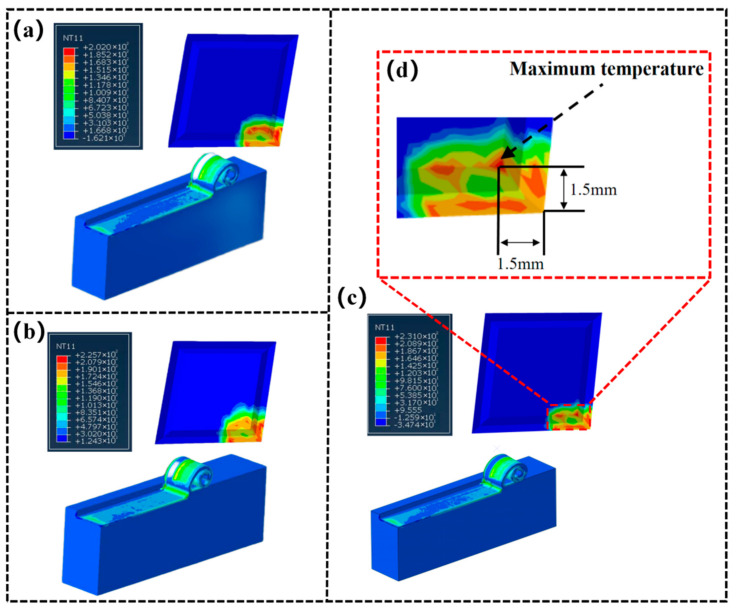
Temperature simulation results of tool and workpiece under different parameters. (**a**) A cutting speed of 113.04 m/min. (**b**) A cutting speed of 150.72 m/min. (**c**) A cutting speed of 188.40 m/min. (**d**) Position of highest milling temperature.

Figure 2 shows the simulation results for the highest temperature position when milling titanium alloy under three parameters. As can be seen from the figure, the highest temperature point generated by milling titanium alloy is located at the front tool face of the milling cutter blade. Its most elevated temperatures were 202, 225.7, and 231 °C, and the highest temperature positions are situated in the tool-chip contact interface, specifically at a distance from the milling tool tip (1.5 mm, 1.5 mm). This is because when the milling cutter removes the titanium alloy, the titanium alloy chip shear deformation generates a large amount of cutting heat, and the cutting heat combined with the friction temperature (between the chip and the front tool surface) leads to the highest temperature in the tool-chip contact area. In this paper, the developed thin-film sensor was embedded in this location for the continuous measurement of the highest temperature value of the milled titanium alloy.

## 3. Sensor Preparation

### 3.1. Sensor Design Solutions

**Figure 3:**
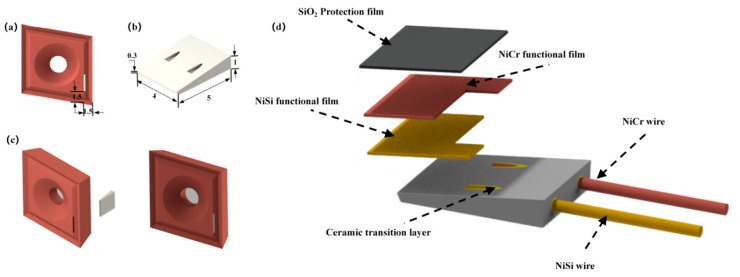
Sensor assembly diagram: (**a**) Position of wedge. (**b**) Substrate of sensor. (**c**) Sensor and blade assembly. (**d**) Structure diagram of new film thermocouple temperature sensor.

In order to avoid damaging the temperature-sensing end of the thin-film thermocouple after frictional wear and to ensure that the sensor can be stably assembled in the milling cutter, the wedge-shaped slot was machined on the front face of the milling cutter using EDM technology, as shown in Figure 3a. The sensor substrate is also designed as a wedge block, as shown in Figure 3b. The length of this wedge block was 5 mm, the width was 4 mm, the maximum height was 1 mm, and the minimum height was 0.3 mm. Figure 3c shows the schematic diagram of the sensor and milling cutter blade assembly, which is fixed to the milling cutter shank by bolts after assembly. Figure 3d shows the structural scheme of the new thin-film thermocouple temperature sensor. The sensor substrate is made of alumina ceramic material, which is designed with two through-holes, both with a diameter of 0.5 mm. It aims to integrate the NiCr-NiSi thin-film compensation lead with this ceramic substrate using static molding technology to form the integrated wires-substrate. In addition, a protective layer film of SiO_2_ was designed above the NiCr-NiSi thermocouple functional film, which was designed to act as an air barrier and prevent the oxidation of the NiCr-NiSi functional film. The NiCr-NiSi thermocouple functional film overlaps the parts of the thermal junction of the sensor [18]. According to the highest temperature location area in the simulation results, the prepared sensor thermal junction area was 4 mm × 1 mm.

### 3.2. Manufacturing of the Integrated Wires-Substrate

**Figure 4:**
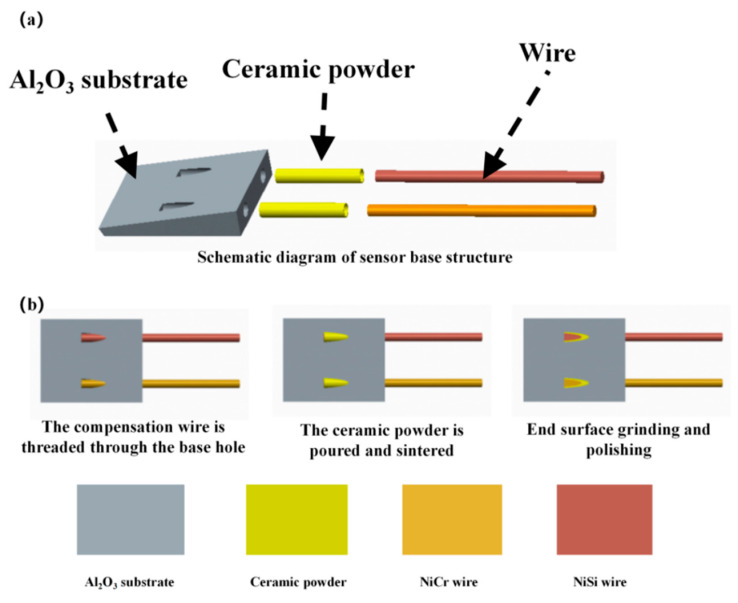
(**a**) Schematic diagram of the sensor substrate structure. (**b**) The integrated wire substrate preparation process.

High purity alumina (99.999%) is a commonly used substrate material for thin-film thermocouple sensors and other sensors [19]. Al_2_O_3_ has the advantages of high strength, chemical stability, corrosion resistance, wear resistance, good insulation, and thermal stability. Therefore, high-purity alumina was selected as the substrate material for the new type of thermocouple temperature sensor. The NiCr-NiSi compensation wire and ceramic substrate were made into an integrated wedge structure using static molding technology, as shown in Figure 4a. The integrated wire substrate preparation process is shown in Figure 4b.

First, the Al_2_O_3_ ceramic substrate and NiCr/NiSi wire were placed into alcohol and then into the ultrasonic cleaner for 10 min, with nitrogen blowing dry. Then, the NiCr and NiSi wires, with a diameter of 0.3 mm, were threaded into the Al_2_O_3_ ceramic substrate reserved hole (diameter of 0.5 mm), with a wire protrusion length of 2–3 mm over the substrate plane. The ceramic powders were poured into the gaps between the wire and reserved and were then placed into a programmable vacuum (high-temperature) furnace with a temperature setting of 600 °C and a holding time of 3 min for high-temperature sintering. After cooling to room temperature, both reached complete fixation. Finally, the integrated wire substrate was mechanically polished to smoothen the working surface of the substrate. Then, the polished substrate was ultrasonically cleaned using acetone, alcohol, and deionized water, with a cleaning time of 10 min, and the surface was blown dry with nitrogen gas.

**Figure 5:**
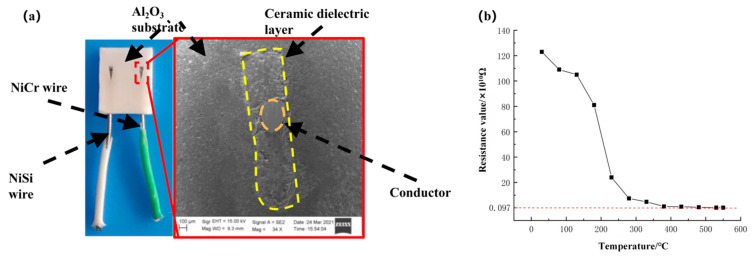
(**a**) SEM image of the bonding surface between the compensating conductor and ceramic substrate. (**b**) Insulation resistance curve of the compensation wire-embedded substrate.

Before preparing the film, to ensure that the end surfaces meet the coating requirements, the surface morphology of the NiCr/NiSi wire-substrate bond was observed with a JEM-2100F scanning electronic microscope (SEM) (Akishima, Japan) after the fabrication of the integrated wire substrate. As shown in Figure 5a, after the polishing process, the ceramic substrate and wire connection were in a good state of bonding, with no apparent defects, and the ceramic substrate surface was a dense and uniform. Using Tektronix-DMM7510 digital multimeter (Beaverton, OR, USA) to measure the resistance value of the NiCr and NiSi wires before and after sintering, the results showed that the NiCr and NiSi wires, before and after sintering, had the same resistance value. Additionally, 600 °C high-temperature sintering does not affect the performance of the wire. After the inline wire substrate was prepared, its insulation properties needed to be tested. We used the ZC-90D high insulation resistance measuring instrument (Shanghai, China), Fluke 9144 dry metering furnace (Everett, WA, USA), to measure the insulation resistance of the wire embedded in the substrate for each temperature stage. We connected the two compensation leads of the embedded substrate to the high resistance meter, placed the substrate inside the chamber of the Fluke 9144 dry metering furnace, set the temperature range from 30 to 550 °C, and adjusted the temperature of the dry metering furnace to measure the resistance value after every 50 °C increase. The resistance curve of the integrated wire substrate insulation test (of the compensation wire) is shown in Figure 5b.

### 3.3. Preparation of Sensor Films

**Figure 6:**
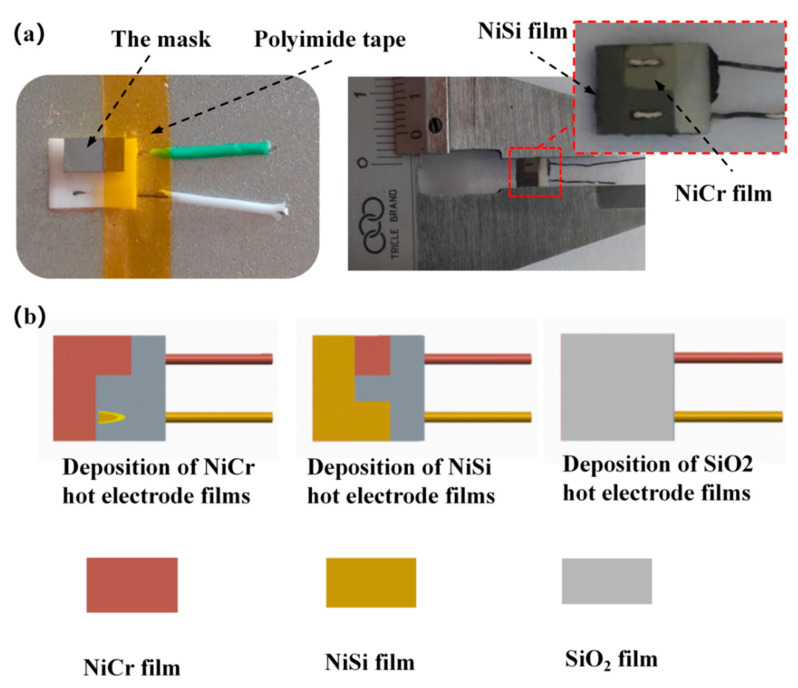
(**a**) Sensor mask. (**b**) Sensor fabrication process.

After the sintering of the ceramic substrate (embedded in the compensation wire) was completed, the functional film of the sensor was prepared. The new thin-film thermocouple temperature sensor functional film mainly includes a NiCr functional film, a NiSi functional film, and a SiO_2_ protective layer film. The cleaned ceramic substrate was fixed onto the particular chuck, and the special chuck was placed into the vacuum magnetron sputtering equipment to prepare the functional thin film; the sensor preparation process is shown in Figure 6b.

The compensation wire in the embedded ceramic substrate and the mask plate were completely fixed on the particular chuck. A NiCr functional film with a thickness of 800 nm was deposited on the substrate using the JZFZJ-500S (Sky Technology Development Co., Ltd., Chinese Academy of Sciences) high vacuum multifunctional composite coating equipment [20] with DC pulse magnetron sputtering technology. After preparing the NiCr film, we removed the NiCr functional film mechanical mask, replacing the NiSi functional film mechanical mask and the target material, and deposited the NiSi functional film to a thickness of 800 nm. The process parameters for the preparation of the NiCr and NiSi functional films are shown in Table 2, and the mechanical masks required for the film preparation are shown in Figure 6a. If the NiCr and NiSi functional films come into contact with air for a long time, this affects film-temperature-measurement performance, and the new thin film thermocouple temperature sensor developed in this paper is directly involved in milling. In order to extend the service life of the sensor, it is necessary to prepare a SiO_2_ protective film on the surface of the functional film to play the role of an anti-oxidation material for wear resistance. The thickness of the SiO_2_ protective film was 800 nm, and the deposition process parameters for the film are shown in Table 3.

**Figure 7:**
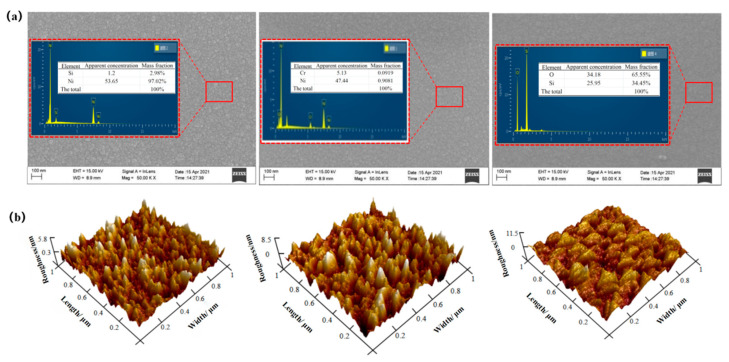
(**a**) Energy spectrum analysis diagram and SEM of the NiCr, NiSi, and SiO_2_ films. (**b**) AFM of the NiCr, NiSi, and SiO_2_ films.

After the preparation of the thin film thermocouple, the essential morphological characterization of each layer was carried out, and the samples prepared in the same furnace were observed and analyzed via a JEM-2100F scanning electronic microscope (SEM) and an energy dispersive spectrometer (EDS) for the microscopic morphology and composition of the film surface. The surface morphology and composition of the NiCr functional film, NiSi functional film, and SiO_2_ protective film are shown in Figure 7a. At a magnification of 50k, the surface grains of the NiCr and NiSi films are dense and flat with uniform distribution and a smooth and dense surface, which ensures good conductivity for the thermal contact of the sensor. The SiO_2_ film surface was uniform and continuous, without evident pinholes and defects, ensuring that the film had excellent insulation properties. The atomic composition ratio of Ni and Cr in the NiCr films was 90.81:9.19, which is close to the atomic ratio of 90:10 for the target composition, and the atomic composition ratio of Ni and Si in the NiSi films was 97.02:2.98, which is close to the atomic ratio of 97:3 for the target composition. The ratio of Si and O atoms in the SiO_2_ films was 34.45:65.55, which is close to the atomic ratio of SiO_2_ of 1:2. From the results, it can be seen that the distribution of each element in the NiCr, NiSi, and SiO_2_ films was uniform, and the quantitative accuracy of the main elements was high, which meets the requirements for film preparation.

Further, a Multimode8 Atomic Force Microscope (Bruker, Berlin, Germany) was used to observe the surface roughness of the prepared functional films, and the results are shown in Figure 7b. The roughness of the NiCr, NiSi, and SiO_2_ films was 5.8, 8.5, and 11.5 nm, respectively, which ensured a uniform coverage for each film layer. The surfaces of the three films have good overall uniformity, continuity, and density, except for individual areas with coarse grains.

### 3.4. Static and Dynamic Calibration of the Sensor

**Figure 8:**
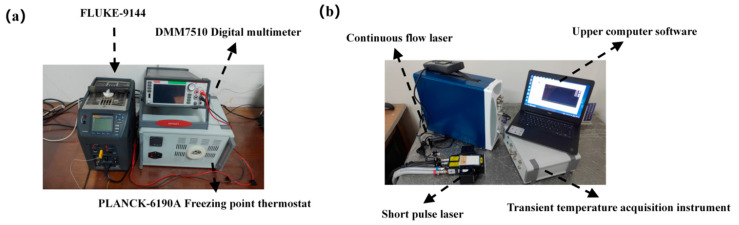
(**a**) Static calibration system. (**b**) Dynamic calibration system.

The Seebeck coefficient and response time are essentially static and dynamic indicators of thin-film thermocouple temperature sensors, respectively [1,21,22]. Therefore, the new thin-film thermocouple temperature sensor was calibrated statically and dynamically. The static calibration system includes a FLUKE-9144 dry metering furnace, PLANCK-6190A freezing point thermostat (Wuhan, China), and a Tektronix-DMM7510 digital multimeter. The structural block diagram of the sensor static calibration system is shown in Figure 8a. The hot node of the thin film thermocouple was placed in a FLUKE-9144 dry metering furnace, and the cold node was placed in a PLANCK-6190A freezing point thermostat and set at a constant 0 °C. The value of the varying thermal potential at different temperatures was recorded with a digital multimeter. We tested the dynamic response time of the developed sensor using the impulse response method. The dynamic calibration system includes a Quantel Laser Ultra 50 short pulse laser (Newbury, UK), transient temperature data acquisition equipment, a computer, etc. The dynamic test system is shown in Figure 8b.

Firstly, we connected the compensation lead of the thin-film thermocouple temperature sensor with the transient temperature data acquisition equipment. Then, we fixed the temperature sensor to the target frame of the test bench after the lead connection was completed, adjusting the red light source of the He-Ne laser to make it focus on the thermal contact position of the thin-film thermocouple temperature sensor (on the target frame) by the frequency doubling crystal. We adjusted the laser source to make it correspond with the red light source of the laser; secondly, we adjusted the voltage value of the laser (by the handle) to 640 V, with a laser energy of 2 mJ. We opened the acquisition software, adjusted the acquisition parameters to 200 k/s, and choose the manual emission mode to output the laser; finally, the emitted laser acted on the film thermal contact area and generated a thermoelectric signal, which was sent to the upper computer software to display the dynamic calibration curve in real-time through the transient temperature acquisition equipment.

**Figure 9:**
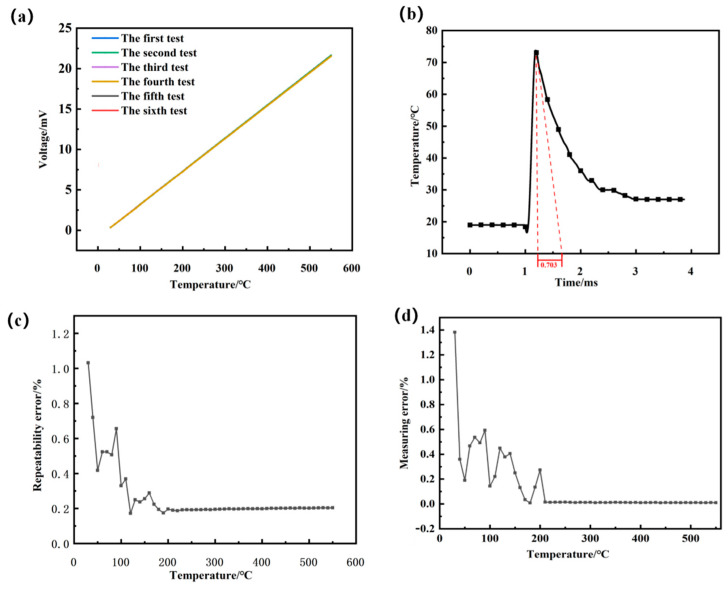
(**a**) Repeatability calibration curve for the thin-film thermocouple temperature sensor. (**b**) Dynamic calibration curve for the thin-film thermocouple temperature measurement blade. (**c**) Repeatability error curve. (**d**) Measurement error curve.

According to the milling range for the titanium alloy temperature, we set the static calibration temperature range to 30~550 °C and adjusted the dry metering furnace to maintain temperature after every 3 min (for every 10 °C rise). In order to ensure that the developed sensor had good repeatability, the sensor was tested for repeatability. The same thin-film thermocouple temperature sensor was subjected to six repeated calibration tests using the above method. Finally, the data obtained from the static calibration were linearly fitted using the least squares method, and the curve is shown in Figure 9a.

(2) E=Sθ+b

where:

E—Thermoelectric potential value;

S—Seebeck coefficient;

θ—Temperature;

b—Intercept.

The average Seebeck coefficient for the six calibrations for the developed sensor was calculated to be 40.72 μV/°C, the maximum nonlinear error was 0.017%, and the linear correlation fitting coefficient sensor reached above 0.999. The sensor was calibrated six times with similar results, and the error calculated for the sensor’s signal data output was 1.39%. The formula for calculating the measurement error is as follows.

*x* = (*A* − *B*) ÷ *B* × 100% (3)

where:

*A* − *B*—The absolute error between the measured value and the true value;

*B*—True Value;

*x*—Measurement error.

The standard deviation is calculated as follows.

(4) $$\sigma =\sqrt{\frac{\sum {\left(x_{i}-x\right)}^{2}}{n-1}}$$

where:

n—Actual number of measurements;

$x_{i}$—Results of each test;

x—Average of multiple measurements;

σ—Standard deviation.

The maximum repeatability error was 1.03%.

(5) $$\delta =\frac{\sigma }{x}\times 100\%$$

The repeatability error curve and the maximum measurement error curve obtained by repeating the calibration for the sensor six times in the same environment are shown in Figure 9c,d.

At 30 °C, the sensor repeatability error was the largest at 1.03%, gradually decreasing with the gradual temperature increase and finally stabilizing at 0.2%. The maximum measurement error also appeared at 30 °C, with a value of 1.41%, and finally stabilized at 0.3% as the temperature gradually increased. The optimal repeatability of the sensor ranges from 36.36% to 100% of the calibration interval.

The impulse response curve of the thin-film thermocouple is shown in Figure 9b. The response time, τ, off the thin-film thermocouple was calculated to be 0.703 ms, with a rapid response time of milliseconds, which meets the requirements of transient milling temperature testing.

## 4. Milling Application Experiment

### 4.1. Milling Temperature Measurement System

**Figure 10:**
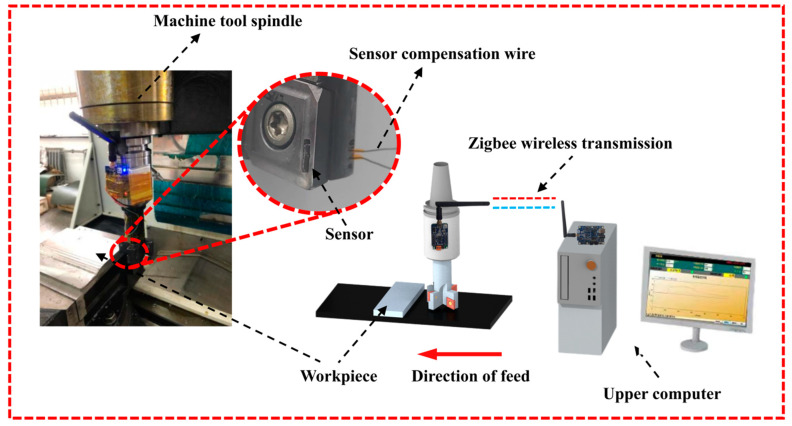
Wireless transient milling temperature measuring device.

Since the thin-film thermocouple temperature sensor does conduct rotational movement with the milling cutter, the temperature signal cannot be transmitted by wired means, so a wireless transient milling temperature testing system was constructed. The temperature test device consists of a new thin-film thermocouple temperature sensor, carbide insert, ATST-type milling cutter (60-C40-H28-150L-4T), wireless transmission system, and host computer software, as shown in Figure 10.

The thin-film thermocouple is mounted on the front face of the milling cutter blade, and the compensation wire is wrapped by insulated heat-shrink tubing and passed through the through-hole of the cutter shank and connected to the wireless transmission module fixed at the cutter shank. The wireless transmission module uses MAX31855 (Maxim Integrated, San Jose, CA, USA) as the temperature acquisition unit to realize the collection of the milling temperature data and uses CC2530 as the main control chip to send the collected temperature data to the upper computer software through ZigBee wireless transmission technology, realizing the real-time display of the milling temperature in the form of the change curve.

**Figure 11:**
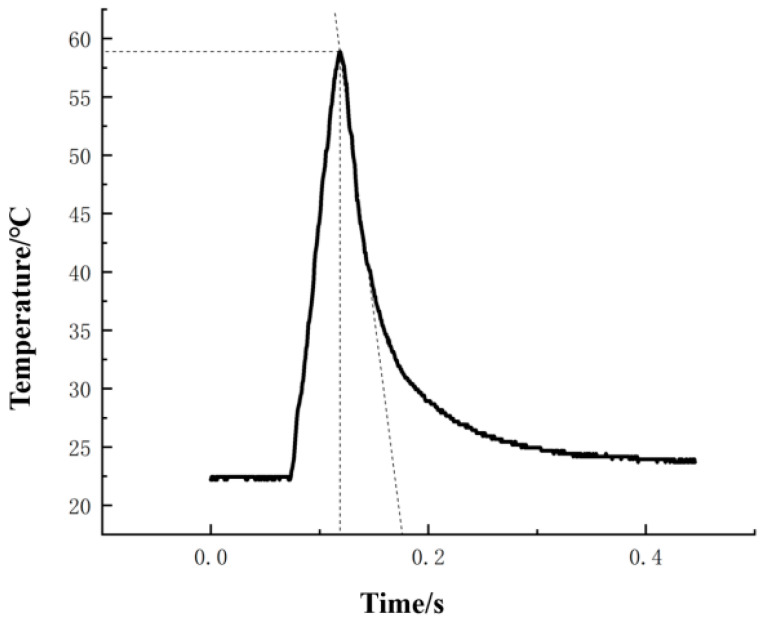
Milling transient temperature profile.

The response time of the system was tested by pre-processing a 2 mm (length) × 1 mm (width) × 1 mm (height) tab on a titanium workpiece and using a cutting speed of 113.04 m/min, a feed of 140 mm/min, and a cutting depth of 0.5 mm as the test parameters. The transient temperature change curve for the milling process was obtained, as shown in Figure 11. The built milling temperature test system can monitor the transient temperature change at the tool-chip contact interface during the milling process within 0.1 s and meet the requirement of real-time milling temperature measurement. The response time of the whole system is lower than the response time of the sensor because the acquisition rate of the MAX31855 chip is around 10 Hz, which limits the response time of the whole test system.

### 4.2. Sensor Performance after Wear and Tear

**Figure 12:**
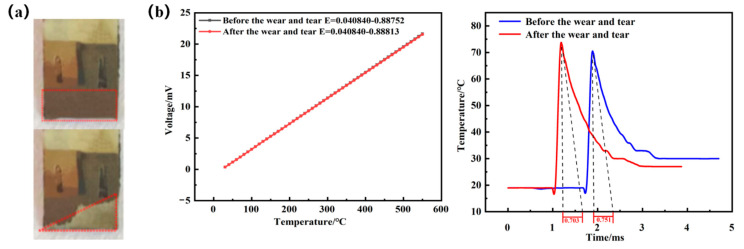
(**a**) Comparison of static calibration curves before and after wear. (**b**) Comparison of dynamic calibration curves before and after wear.

The cutting speed was maintained at 113.04 m/min, with a feed of 140 mm/min and a cutting depth of 0.5 mm, and the milling cycle was set up to continuously mill a titanium alloy piece (160 mm × 140 mm × 50 mm) for 30 min. The wear pattern of the sensor was obtained and is shown in Figure 12a. Due to a machining error, there is a small gap between the embedded sensor and the tool holder. A small number of chips will enter the gap during the machining process to cause wear to the sensor. As seen from the figure, after the milling test, the wear area of the sensor thermal junction was about one-half of the original area. In order to investigate whether the sensor’s performance would be affected after wear, it was re-calibrated for static and dynamic calibration, and the static and dynamic calibration results are shown in Figure 12b.

The analysis of the static and dynamic calibration data before and after the wear of the thin-film thermocouple temperature sensor thermal junction shows that the difference between the Seebeck coefficient of the sensor before and after wear is not significant. The change is 0.01 μV/°C, the maximum rate of change of the sensor temperature measurement node is 0.2%, and the time constants are 0.703 ms and 0.751 ms. The time constants are of the same order of magnitude, and the values are close. Therefore, it can be shown that the thin-film thermocouple temperature sensor thermal node wear will not change its dynamic and static temperature measurement performance, and the milling test can meet the requirements of continuous temperature measurement.

### 4.3. Milling Temperature Simulation Verification

**Figure 13:**
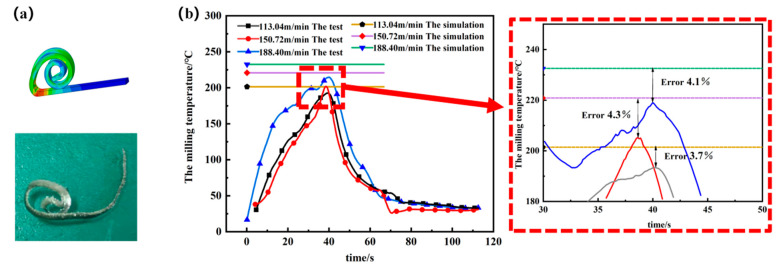
(**a**) Comparison of the milled and simulated chip shapes. (**b**) Milling test temperature and the simulation maximum point comparison.

To ensure that the simulation parameters are consistent with the parameters of the titanium alloy field test, we set the feed of the milling machine to 200 mm/min and the cutting depth to 0.5 mm, and choose three different cutting speeds: 113.40, 150.72, and 188.04 m/min for the titanium alloy milling test, respectively. The field test temperature data were compared with the maximum value of the milling temperature obtained from the simulation. Figure 13a shows the obtained titanium alloy chips compared with the simulated chips for their morphology, and Figure 13b shows the titanium alloy milling test temperature compared with the simulated maximum point.

As can be seen from the figure, the titanium alloy simulation chip and milling test chip shape are the same, and the milling temperature continues to rise when the milling cutter first cuts into the titanium alloy. After a while, the temperature reaches the highest value. When the milling cutter cuts the titanium alloy, the temperature slowly decreases and tends to room temperature. By analyzing the error between the highest value of the simulation and the highest value of the titanium alloy test, it can be seen that the maximum error is 4.3%, which is within the permissible range. From the results of the chip comparison and the highest point temperature comparison (for milling), it can be seen that the designed wireless temperature measurement device for milling, based on a thin-film thermocouple temperature sensor in the tool-chip contact area, has practicality and temperature measurement accuracy.

### 4.4. Milling Temperature Data Analysis

**Figure 14:**
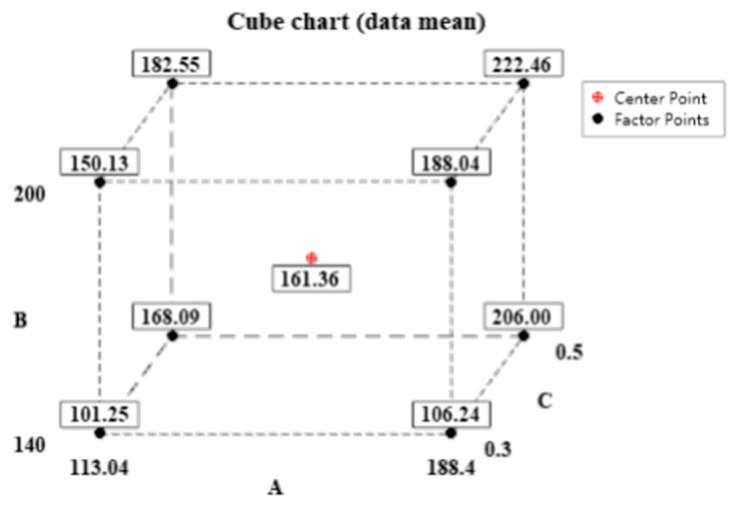
Cubic diagram.

**Figure 15:**
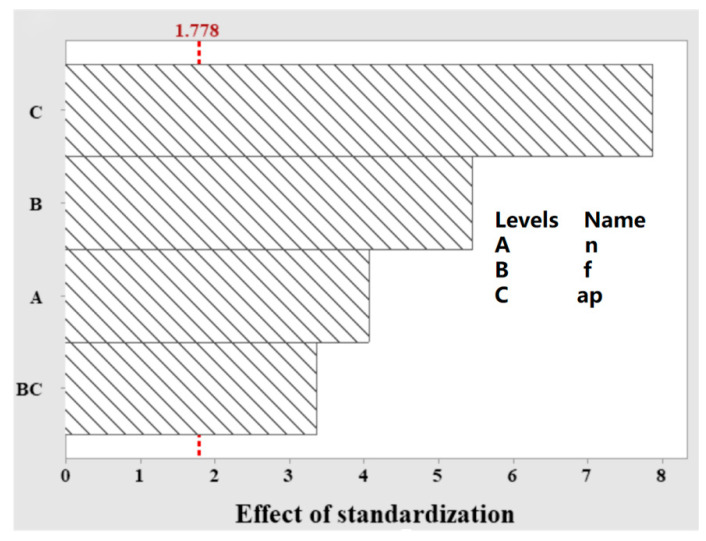
Pareto plot of normalized effects.

A DOE (design of experiment) test with three factors and two levels, containing a center point, was designed using cutting speed, feed, and cutting depth to investigate the effect of milling parameters on the maximum temperature of milling. The factor levels are shown in Table 4. In order to obtain comprehensive test information, the above milling temperature measurement device was used to perform titanium alloy milling tests with the whole factorial test method, with a total of nine tests. Figure 14 shows the cubic diagram, and the test combinations and results are shown in Table 5. The maximum milling temperature is used as the response R to the data at a confidence level of 0.05 using a stepwise method to analyze the designed factors to ensure that the optimal solution for temperature regulation is found. The ANOVA results are shown in Table 6. The P-value for the whole model is less than 0.05, which proves that the model is significantly valid. The milling parameters with the lowest temperature were as follows. A cutting speed of 113.04 m/min, a feed of 140 mm/min, and a cutting depth of 0.3 mm, which have significant effects on milling temperature in the order of cutting depth, feed, cutting speed, cutting depth*feed, for which the P-values were less than 0.05. Table 7 shows the model summary table, which has an R-sq of 96.76%, indicating a good fit of the model to the data, and from the Pareto plot of the standardized effects in Figure 15, it can be seen that the model has an absolute value of 1.778 for the effects, with all standardized effects exceeding this value, so the regression model is valid.

**Figure 16:**
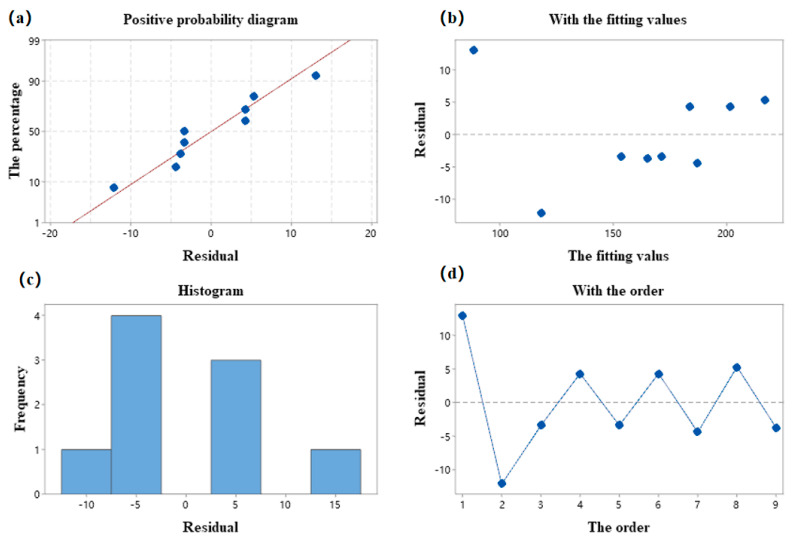
(**a**) Normal probability plot. (**b**) Plot with fitted values. (**c**) Plot with fitted values. (**d**) Plot with fitted values.

Figure 16 shows the four-in-one residual plot of this regression model. All points in the positive terrestrial probability plot approximate a straight line, and the residuals are consistent with normality. In the residual versus fitted value plot and the residual versus order plot, all points are evenly distributed on both sides of the 0 point, which is normal. The optimized regression equation is as follows.

Temperature = −409.1 + 0.4005 *n* + 2.336 *f* + 998 *ap* − 4.16 *f* * *ap* (6)

## 5. Conclusions

A new thin-film thermocouple sensor was developed for continuous temperature measurement during the milling process. The sensor was applied to a titanium alloy milling process to obtain a temperature change curve for the tool-chip contact area during the milling process. After the milling experiments, the effect of thermal junction wear on the sensor’s performance was investigated. The research in this paper provides a new method for the real-time cutting measurement of rotary-type tools. The main findings are as follows.

1. The ceramic sintering technique embeds the compensation lead into the alumina substrate, which realizes the structural integration of the lead and the sensor substrate. The SEM image shows that the compensation lead is well bonded to the substrate with a dense and flat surface, which meets the requirements for thin film preparation, and is mounted into the tool with a wedge-type structure, effectively improving the stability of the sensor. The insulation of the substrate was tested in a high-temperature environment from 30 to 550 °C, and the test results met the insulation requirements;
2. The nanocomposite multilayer films were prepared on the substrate by a magnetron sputtering technique and characterized for their microscopic morphology. The results showed that the prepared films meet the requirements for use. The sensor film was characterized, and the sensor was calibrated statically and dynamically. The results showed that the developed temperature sensor has a sensitivity of 40.72 μV/°C, a maximum repeatability error of 1.38%, and a dynamic response time of 0.703 ms;
3. The sensor was applied to a titanium alloy milling process to obtain the temperature variation profile in the tool-chip contact area during milling. The accuracy of the simulation model was verified by comparing the highest value for the chip and temperature in the experiment using the same milling parameters as the simulation. After 30 min of continuous milling, the wear area of the thermal junction of the sensor was about one-half of the original area. However, the thermal junction wear did not affect the continuous temperature measurement;
4. A three-factor, two-level test containing one center point was designed using DOE for the cutting speed, feed, and cutting depth. Furthermore, the experimental results were analyzed by ANOVA. In order to reduce the temperature of the milling process, a smaller cutting depth should be used from the perspective of single-factor adjustment, and a smaller cutting depth and feed should be used from the perspective of interactive factor adjustments. The regression equation for the temperature and milling parameters was obtained, and the R-sq of this regression model was 96.76%.

## Figures and Tables

**Table 1:** JC model parameters for TC4 material.

The Material Properties	*A*	*B*	*C*	*n*	m
Value	870	990	0.008	1.01	1.4

**Table 2:** Preparation technological parameters of the NiCr/NiSi thin film.

Vacuum/Pa	Work Pressure/Pa	Ar Flow/sccm	O_2_ Flow/sccm	Sputtering Power/W
6.0 × 10^−4^	0.7	20	100	50
6.0 × 10^−4^	0.7	20	100	48

**Table 3:** Preparation technological parameters of the SiO_2_ thin film.

Vacuum/Pa	Work Pressure/Pa	Ar Flow/sccm	O_2_ Flow/sccm	Sputtering Power/W	Sputtering Time/h
1.0 × 10^−3^	0.7	20	10	150	3

**Table 4:** Factor and level information.

Factor	Number of Levels	Value
Cutting speed (*n*)	2	113.04, 188.40
Feed (*f*)	2	140,200
Cutting depth (*ap*)	2	0.3,0.5

**Table 5:** Comprehensive test results.

Serial Number	Cutting Speed/(m/min)	Feed/(mm/min)	Cutting Depth/mm	Maximum Temperature/°C
1	188.40	200	0.3	188.04
2	113.04	140	0.5	168.09
3	188.40	140	0.5	206.00
4	113.04	200	0.5	182.55
5	188.40	200	0.5	222.46
6	150.72	170	0.4	161.36
7	188.40	200	0.3	188.04
8	113.04	140	0.5	168.09
9	188.40	140	0.5	206.00

**Table 6:** ANOVA analysis of variance result table.

Source	Free Degree	Adj SS	Adj MS	F-Value	*p*-Value
Model	4	13141.8	3285.44	29.85	0.003
linear	3	11897.8	3965.92	36.03	0.002
*n*	1	1821.7	1821.66	16.55	0.015
*f*	1	3264.3	3264.32	29.66	0.006
*ap*	1	6811.8	6811.78	61.88	0.001
Interaction of factors	1	1244	1244.01	11.3	0.028
*F* * *ap*	1	1244	1244.01	11.3	0.028
Error	4	440.3	110.07		
Total	8	13582.1			

**Table 7:** Model summary table.

S	R-sq	R-sq (Adjustment)	R-sq (Forecast)
10.4917	96.76%	93.52%	79.12%
